# Renal allograft-related inflammation complicated by acute coronary syndromes: A case report

**DOI:** 10.1097/MD.0000000000028205

**Published:** 2021-12-30

**Authors:** Rainer U. Pliquett, Andrea Tannapfel, Sait S. Daneschnejad

**Affiliations:** a Department of Nephrology, Diabetology and Endocrinology, University Hospital Leipzig (RUP until June 2006); b Department of Nephrology & Diabetology, Carl-Thiem Hospital Cottbus, Cottbus, Germany; c Institute of Pathology, University Hospital Leipzig, Leipzig, Germany; d Institute of Pathology, Ruhr-University Bochum, Bochum, Germany; e Department of Cardiology, Helios Hospital Schkeuditz, Schkeuditz, Germany.

**Keywords:** acute coronary syndrome, case report, coronary artery disease, inflammation

## Abstract

**Introduction:**

Persistent systemic inflammation is considered to be predictive for future cardiovascular events. Here, in a patient with pyelonephritis of his failed renal allograft, consecutive coronary angiograms proved that coronary artery disease progressed within 3 weeks, when infection was uncontrolled.

**Patient concerns:**

A 52-year-old male type 2 diabetic with a failed renal allograft suffering from hematuria, leukocyturia, and chest pain at rest was hospitalized.

**Diagnoses:**

An acute coronary syndrome in presence of pyelonephritis was diagnosed. Besides pyelonephritis, the histological examination of the kidney transplant revealed signs of chronic rejection and the presence of a renal cell carcinoma *in situ*.

**Interventions:**

A percutaneous coronary intervention was performed, and an elective surgery for allograft removal was scheduled. However, within 5 weeks after discharge, two more surges of infection coincided with episodes of unstable angina.

**Outcomes:**

Once the renal allograft has been removed, systemic inflammation was contained. The patient was not re-hospitalized for acute-coronary syndrome within the next 12 months.

**Conclusion:**

Surges of systemic inflammation due to infection were paralleled by instability of coronary plaques as documented by repeat coronary angiograms.

## 1 Introduction

C-reactive protein (CrP), a surrogate of systemic inflammation, is considered to be a predictor for cardiovascular risk in the general population[^1^,^2^,^3^] and for patients with acute myocardial infarction^[4]^ or unstable angina.^[5]^ However, it remains unclear, whether or not CrP plays an active role in atherothrombosis or solely represents an epiphenomenon in acute coronary syndrome.^[6]^

Here we report a patient with end-stage renal disease due to a failed, super-infected renal allograft hospitalized for pyelonephritis and repeat episodes of acute coronary syndrome. The role of systemic inflammation for coronary artery disease outcomes is discussed.

## 2 Case presentation

A 52-years’ old, male patient of White ethnicity was admitted to a nephrology department of a tertiary referral hospital presenting with hematuria, leukocyturia and oligo-anuria in the presence of a failed renal allograft. 12 hours before admission, the patient had chest pain at rest. However, no action was taken. Apart from end-stage renal disease due to diabetic nephropathy and coronary artery disease, comorbidities included type-2 diabetes with a low hemoglobin A1c of 5.3%, android obesity with a body mass index of 33.6 kg/m^2^, a poorly controlled arterial hypertension, dyslipoproteinemia with hypertriglyceridemia and a low high-density lipoprotein cholesterol. In addition, he suffered from rheumatic arthritis, secondary hyperparathyroidism, an epoetin-resistant anemia (hemoglobin on admission: 5.4 mmol/L, ferritin: 1302 ng/mL). The patient received a renal allograft of a deceased donor 4 years earlier after 7 years on hemodialysis. Hemodialysis had to be resumed 6 months before hospitalization.

As for medical therapy, the patient received an intensive insulin treatment in addition to standard cardiovascular oral medications including angiotensin converting enzyme inhibitor, statin, low-dose aspirin, beta-blocker agent, and subcutaneous epoeitin alpha.

The patient had a history of multiple percutaneous coronary interventions for obstructive coronary artery disease in the preceding 6 years. Before hospitalization, an echocardiographic examination demonstrated a preserved left-ventricular systolic function.

**Figure 1:**
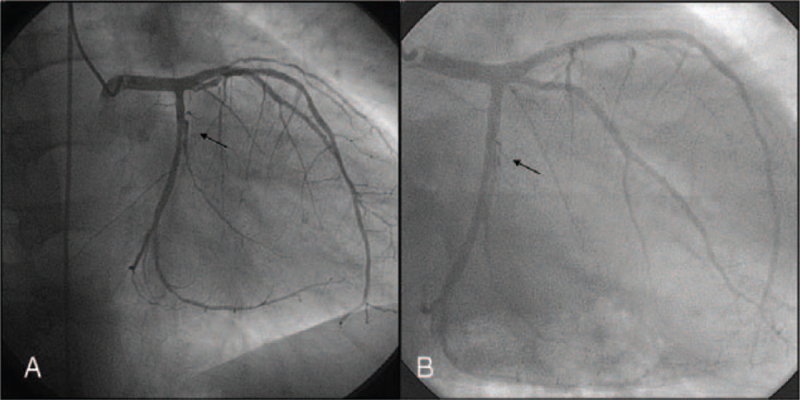
Coronary angiogram of the left coronary artery with left anterior descendant and left circumflex coronary arteries (A) at index hospitalization, (B) 3 weeks later on second hospitalization following episodes of unstable angina. Arrows indicate the position before and after a newly developed culprit lesion within a branch of the LCX causing the latter episode of unstable angina.

**Figure 2:**
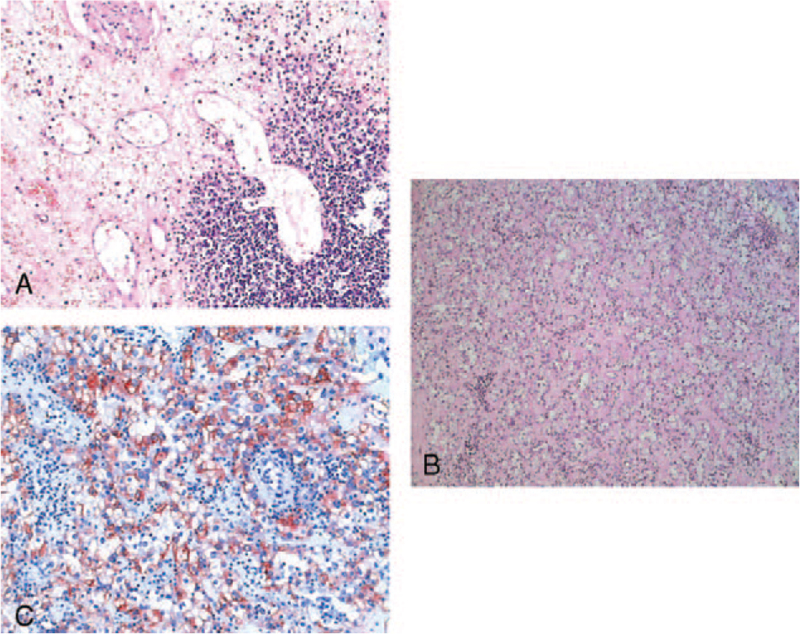
(A) Severe chronic inflammation of the graft with dilated cysts and fibrosis. (Hematoxylin-Eosin; original magnification ×20). (B) Moderately differentiated renal cell carcinoma (Hematoxylin-Eosin; original magnification ×20). (C) Immunohistochemical visualization of cytokeratin within the tumor (brown reaction product: anti-cytokeratin [1/400, Clone; MNF 116; Dako], antibody for 2 h at room temperature; original magnification ×25).

On admission, physical examination was unremarkable. Troponin T was elevated slightly (0.15 μg/L), creatinine kinase was normal. CrP (155.1 mg/L), and procalcitonin (1.38 ng/mL) was elevated. The electrocardiogram (ECG) remained unchanged showing preexisting T-wave inversions in I and aVL. Transthoracic echocardiography revealed a moderate left-ventricular hypertrophy and a new inferoseptal and basoapical hypokinesia. Consecutively, a percutaneous coronary intervention with bare-metal stent deployment into a right-coronary-artery stenosis was performed. At that time, the left coronary artery including left anterior descendent and circumflex artery had no stenosis (Fig. 1A). For a urinary tract infection, intravenous ciprofloxacin was administered, CrP leveled off at 52.1 mg/L. As the urinary-tract infection persisted, an elective renal allograft-removal surgery was scheduled one month later, once dual antiplatelet therapy would be switched to a long-term antiplatelet monotherapy. However, 3 weeks later, the patient was readmitted for acute coronary syndrome. CrP was 140.7 mg/L. The ECG revealed a new significant Q wave in III and prominent T waves in the precordial leads V2 and V3. Transthoracic echocardiography showed a new akinesia of the posterior wall, a subsequent coronary angiography revealed a new unstable plaque in the first branch of the left circumflex coronary artery (Fig. 1B) being absent 3 weeks earlier (Fig. 1A). There, a bare-metal stent was deployed directly. To control the infection, the allograft-removal surgery was performed as an emergency one week after readmission, despite an ongoing dual antiplatelet therapy. The histological analysis revealed abscess formation and a prevalent vasculitis as a sign of chronic vascular rejection (Fig. 2A). Unexpectedly, a moderately differentiated renal cell carcinoma (G2, pT1a, pN0, L0, V0) with a diameter of 2.5 cm was found (Fig. 2B and C). The tumor was restricted to the renal transplant showing no signs of invasion. Abdominal and thoracic computed tomographies revealed no metastasis to lymph-nodes or distant organs. The patient remained in a stable condition before and after surgery.

Within 6 days following emergency allograft-removal surgery, the patient experienced a third episode of troponin-positive, unstable angina when inflammation-related parameters rose again (maximum CrP: 206.2 mg/L). Creatinine kinase remained normal. Echocardiography showed no change, in spite of new negative T waves in the precordial ECG leads V4 – V6. Under conservative therapy including intravenous heparin and antibiosis, the patient remained in a stable condition. The wound infection consolidated. Within 10 days after surgery, the overall condition improved. Plasma albumin increased from 25.5 g/L to 32.9 g/L, CrP leveled off at 64.7 mg/L. 4 months following surgery, the physical examination, the sonographic examination of arteriovenous fistula for hemodialysis access and a scheduled coronary angiography were unrevealing. The patient was not re-hospitalized for acute-coronary syndrome within the 12 months following discharge.

## 3 Discussion

In the patient presented here, peak systemic inflammation coincided with acute coronary syndromes. Once the renal allograft ceased to function due to pyelonephritis and due to chronic allograft rejection, increased systemic inflammation may have facilitated the occurrence of plaque ruptures leading to acute coronary syndromes. This view is supported by sequential coronary angiograms within 3 weeks. As soon as systemic inflammation was consolidated, the patient remained event-free, as far as acute coronary syndromes are concerned.

After kidney transplantation, medical immunosuppressive therapy may predispose to infections. Once the main source of infection has been identified, other possible secondary sources of infection such as infective endocarditis need to be ruled out, if systemic inflammation still persists. In the present case, except for pyelonephritis within the renal allograft, no secondary source of inflammation was found.

As a limitation, no cause–consequence relationship between inflammation and acute coronary syndromes may be established from a single case. However, the timely coincidence points to the possibility of a contribution of systemic inflammation to the occurrence of cardiovascular events. Coronary plaque rupture is known to be facilitated by pro-inflammatory cytokines.^[7]^ Besides local inflammatory processes leading to coronary-plaque instability, a more generalized inflammatory response may affect the whole coronary tree. There, CrP synthesized in the liver upon interleukin-6 stimulation, represents a candidate mediator linking inflammation with occurrence of acute coronary syndromes.^[8]^ In an animal model of atherosclerosis, CrP transgene expression accelerated aortic atherosclerosis,^[9]^ whereas therapeutic inhibition of CrP improved outcome of experimental myocardial infarction.^[10]^ Functional studies on neutrophils and human endothelial cells indicate that monomeric CrP, but not pentameric or native CrP, leads to neutrophil-granulocyte^[11]^ and to endothelial-cell activation.^[12]^

Increased systemic inflammation adversely affects mortality in patients with chronic kidney disease, stage 3 or 4 of.^[13]^ However, a reduced renal function correlates with overall and cardiovascular mortality as well.^[14]^ Future research needs to determine the individual role of uremic toxins and of inflammatory parameters for cardiovascular outcomes. To differentiate the possible role of CrP on acute coronary-artery disease progression, adsorptive techniques may be employed to therapeutically lower plasma CrP in cardiovascular high-risk patients on an individual basis.^[15]^

## 4 Conclusion

From this case, a contributory role of inflammation on acute progression of coronary heart disease is assumed. Anti-inflammatory strategies need to be considered in patients with therapy-resistant systemic inflammation to avoid cardiovascular complications.

## Acknowledgments

The authors thank the patient for providing his consent for the publication.

## Author contributions

RUP and SSD were attending doctors of the patient. RUP drafted the paper. SSD gave critical input. AT critically contributed to contents concerning the histological specimen. All authors contributed to the discussion and approved the content of this manuscript.

**Conceptualization:** Rainer U. Pliquett, Andrea Tannapfel, Sait S. Daneschnejad.

**Data curation:** Rainer U. Pliquett, Andrea Tannapfel, Sait S. Daneschnejad.

**Formal analysis:** Rainer U. Pliquett, Andrea Tannapfel, Sait S. Daneschnejad.

**Investigation:** Rainer U. Pliquett, Andrea Tannapfel.

**Methodology:** Rainer U. Pliquett, Andrea Tannapfel, Sait S. Daneschnejad.

**Project administration:** Andrea Tannapfel.

**Resources:** Rainer U. Pliquett, Andrea Tannapfel, Sait S. Daneschnejad.

**Software:** Rainer U. Pliquett.

**Supervision:** Sait S. Daneschnejad.

**Validation:** Rainer U. Pliquett, Andrea Tannapfel, Sait S. Daneschnejad.

**Visualization:** Andrea Tannapfel.

**Writing – original draft:** Rainer U. Pliquett.

**Writing – review & editing:** Rainer U. Pliquett, Andrea Tannapfel, Sait S. Daneschnejad.
